# Effects of Mannitol on Rumen and Fecal Microbiome, Immune Function, and Antioxidant Capacity of Sunit Sheep

**DOI:** 10.1002/fsn3.72527

**Published:** 2026-11-01

**Authors:** Min Li, Li Tao, Yang Li, Chao Wang, Fugui Yu, Jian Gong, Min Xiao

**Affiliations:** ^1^ College of Life Sciences and Technology, Inner Mongolia Normal University Hohhot Inner Mongolia Autonomous Region China; ^2^ Inner Mongolia Academy of Agriculture and Animal Husbandry Science Hohhot Inner Mongolia Autonomous Region China; ^3^ Sunit Right Banner Jufeng Zhengbei Feed Technology Co., Ltd. Saihantal Inner Mongolia Autonomous Region China; ^4^ Key Laboratory of Biodiversity Conservation and Sustainable Utilization for College and University of Inner Mongolia Autonomous Region Hohhot Inner Mongolia Autonomous Region China

**Keywords:** antioxidant capacity, fecal microbiota, immune function, mannitol, rumen microbiota, sheep

## Abstract

This study evaluated the effects of dietary supplementation with uncoated or coated mannitol on growth performance, rumen fermentation, rumen/fecal microbial communities, and serum immune and antioxidant indices in Sunit sheep. Twenty‐four healthy 7–8‐month‐old male Sunit sheep (28.84 ± 7.08 kg) were randomly assigned to three groups (*n* = 8/group): control (CON; corn flour without mannitol), uncoated mannitol (UM; corn flour + 2 g/kg uncoated mannitol), and coated mannitol (CM; corn flour + 2.6 g/kg coated mannitol). After a 7‐day dietary adaptation period, all sheep were fed a uniform total mixed ration (TMR) for a 14‐day formal experimental period. The results indicated that neither uncoated nor coated mannitol supplementation significantly affected the average daily gain (ADG) (*p* > 0.05). However, both treatments improved the gastrointestinal microbial structure and altered rumen fermentation, as evidenced by increased ruminal pH and decreased short‐chain fatty acid (SCFA) concentrations (*p* < 0.05). For serum biochemistry, the UM group had higher total protein (TP), albumin (ALB), and creatinine (CREA) levels, and lower urea (UREA) levels (*p* < 0.05); the CM group had higher TP and ALB, and lower UREA levels (*p* < 0.05). Additionally, the CM group exhibited increased IL‐4, IgA, C3, SOD, and GSH‐Px levels, and decreased TNF‐β, IFN‐γ, and MDA levels (*p* < 0.05). Correlation analysis revealed a strong negative correlation between serum TP and fecal *norank_o__Gastranaerophilales* abundance (*p* < 0.01), and a negative correlation between IL‐4 and ruminal *Christensenellaceae_R‐7_group* abundance (*p* < 0.05). In conclusion, both the mannitol forms reshaped ruminal and intestinal microbial structures and improved serum indices, with coated mannitol associated with more consistent improvements in immune and antioxidant indices.

## Graphical abstract

This graphical abstract illustrates the effects of uncoated and coated mannitol supplementation on ruminal and fecal microbiomes, serum immune indices, antioxidant status, and metabolism in Sunit sheep. Neither uncoated nor coated mannitol altered growth performance. Both forms of mannitol reshaped ruminal and fecal microbiota, increased ruminal pH, and modulated short‐chain fatty acid production. Meanwhile, supplementation with either form of mannitol improved serum nutritional and metabolic parameters, regulated serum immune factors, and enhanced the antioxidant capacity of Sunit sheep.

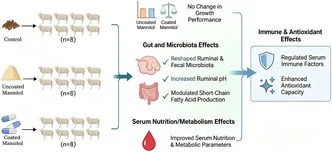

## 1 Introduction

Prebiotics are bioactive substances selectively utilized by beneficial microorganisms in the host gastrointestinal tract (Khawar et al. 2025). Previous studies have confirmed that prebiotics modulate gastrointestinal microbiota structure (Alabi et al. 2025), enhance immune function by improving microbial balance (Wang et al. 2021; Yang et al. 2021), ameliorate lipid metabolism (Wang et al. 2022), showing great application value in ruminant research and production. Notably, carbohydrate‐based prebiotics have attracted extensive attention due to their abundant sources, compatibility with ruminant physiology, and stable effects (Zhu et al. 2025). For instance, dietary inulin supplementation in weaned kids tended to elevate P70S6K (mTOR‐downstream) gene expression, increased average daily gain, and improved intestinal immune function; lower methane production was observed in the in vitro rumen fermentation assay (Yuan et al. 2023). Mannan‐oligosaccharides (MOS) alleviate inflammation and enhance intestinal immunity in newborn goats (Yang et al. 2021), while galacto‐oligosaccharides (GOS) improve growth performance and rumen fermentation in pre‐weaning Holstein calves (Chang et al. 2022). Collectively, dietary supplementation with prebiotics to regulate gut microbiota has become a key strategy for maintaining ruminant health and promoting sustainable livestock farming.

In conventional breeding, antibiotics effectively prevent intestinal diseases and improve growth performance. However, long‐term unreasonable application disrupts rumen and intestinal microbial balance, induces drug resistance, reduces nutrient absorption, and leaves residues in animal products, thereby endangering human health and violating the concept of sustainable breeding (Khan et al. 2025; Li, Yousif, et al. 2019; Manzoor et al. 2025). Mannitol, a natural hexahydric alcohol widely distributed in microorganisms and plants is considered a feed additive with antibiotic‐reduction potential, yet its physiological regulatory effects in ruminants remain to be explored (Cherian et al. 2025), As a poorly absorbed carbohydrate derivative, most ingested mannitol enters the large intestine and is fermented by gut microbiota into short‐chain fatty acids (SCFAs), exhibiting prebiotic functions that have been verified in monogastric animals (Jin et al. 2019). For example, dietary D‐mannitol promotes cecal bacterial proliferation, improves nitrogen utilization and prolongs digesta mean retention time (MRT) in rabbits (Min et al. 2013), and reduces cecal pH and enhances mineral absorption in rats (Xiao et al. 2013). Our previous study revealed that dietary uncoated mannitol regulates gastrointestinal microbiota and improves immune and antioxidant capacity in grazing Mongolian sheep (Min et al. 2025). However, uncoated mannitol is susceptible to degradation by ruminal microbes, which limits its delivery to the hindgut. Whether mannitol can be effectively utilized by hindgut microorganisms and sustain prebiotic functions in ruminants remains unclear. Furthermore, gastrointestinal microbial composition and nutrient metabolism vary among sheep breeds, implying potential breed‐specific responses to mannitol supplementation. To explore whether coating alleviates ruminal degradation and optimizes the regulatory effects of mannitol, the present study compared uncoated and coated mannitol in Sunit sheep, focusing on ruminal and fecal microbiota, serum biochemistry, immune status and antioxidant profiles. We hypothesized that coated mannitol would exhibit greater hindgut prebiotic potential than uncoated mannitol by resisting ruminal breakdown, which may provide a preliminary reference for ruminant healthy feeding strategies.

## 2 Materials and Methods

This study was conducted in compliance with the approval and ethical guidelines of the Experimental Animal Care Committee of Inner Mongolia Normal University in Hohhot, China, with the authorization NO. IMNU‐2025‐025.

### 2.1 Animals and Feeding Management

This experiment was conducted at Zhengbei Ranch in Saihantal Town, Sonid Right Banner, Xilingol League, China. A total of 24 healthy male Sunit sheep aged 7–8 months with an average body weight of 28.84 ± 7.08 kg were selected as experimental subjects and randomly assigned to three groups (*n* = 8 per group). The diary treatments were defined as follows: the control group (CON) was fed a basal corn flour diet without any mannitol supplementation; the uncoated mannitol group (UM) was fed corn flour supplemented with 2 g/kg mannitol (Aladdin Biochemical Technology Co. Ltd., Shanghai, China); the coated mannitol group (CM) was fed corn flour supplemented with 2.6 g/kg coated‐mannitol product, consisting of 2 g/kg active mannitol (uncoated‐equivalent) and 0.6 g/kg coating material. Thus, both groups received an identical active mannitol dose of 2 g/kg in the diet. The supplementation dosage of mannitol in this study was determined based on previous relevant reports (Min et al. 2025). During the experiment, each sheep was first fed 100 g of the corresponding treatment corn flour once daily at 09:00. After complete consumption of the corn flour was confirmed, a total mixed ration (TMR) was provided uniformly. Supplementary feeding of the ration was conducted at 15:00 daily, and free access to water was ensured for all sheep throughout the day. The total experimental period lasted 21 days, consisting of a 7‐day adaptation phase followed by a 14‐day formal experimental phase.

### 2.2 Collection of Blood, Ruminal Fluid and Fecal Samples

Blood, ruminal fluid and fecal samples were collected on day 21 in the experiment. Blood was sampled from the jugular vein and kept at room temperature for 30–40 min, and then centrifuged at 1800 × *g* for 20 min to isolate serum before snap‐freezing in liquid nitrogen. Around 50 mL ruminal fluid was collected by oral‐esophageal stomach tube to reduce saliva contamination, filtered with four layers of sterile cheesecloth and stored in liquid nitrogen for microbiome analysis. Fecal pellets were rectally collected and instantly frozen in liquid nitrogen. All samples were delivered to the lab and stored at −80°C prior to analysis. The sampling protocol was consistent with our previous work.

### 2.3 Body Weight and Ruminal Fermentation Parameter Measurements

Body weights of animals were recorded pre‐feeding at trial commencement and reweighed immediately before sampling on Day 21. Average daily gain (ADG) was calculated from these two weight records. At trial end, ruminal fluid was filtered through four layers of cheesecloth, with pH rapidly measured with a portable pH meter (AS‐211; Horiba Ltd., Kyoto, Japan). SCFAs were quantified by high‐performance liquid chromatography fitted with an Agilent TC‐C18(2) column (250 × 4.6 mm, 5 μm) and Hitachi 5400 FL detector (Hitachi High‐Technologies Corporation, Kyoto, Japan), following Liang et al. (2017). Additionally, ruminal NH_3_–N concentration was determined using the phenol‐hypochlorite colorimetric (Berthelot) method (Broderick and Kang 1980).

### 2.4 DNA Extraction and PCR Amplification

Microbial genomic DNA was isolated from ruminal fluid and feces using the FastDNA SPIN Kit for Soil (MP Biomedicals, Solon, OH, USA) per manufacturer guidelines. DNA integrity was verified by 1% agarose gel electrophoresis, with its concentration and purity determined with a NanoDrop 2000 UV‐vis spectrophotometer (Thermo Scientific, Wilmington, USA). The V3–V4 hypervariable region of the bacterial 16S rRNA gene was amplified with primer set 338F/806R on an ABI GeneAmp 9700 thermal cycler (ABI, CA, USA). Three technical PCR replicates were run for each sample to improve reproducibility. Amplicons were resolved on 2% agarose gels, purified using the AxyPrep DNA Gel Extraction Kit (Axygen Biosciences, Union City, CA, USA), and quantified on a Quantus Fluorometer (Promega, USA).

### 2.5 Illumina MiSeq Sequencing and Data Analysis

Purified amplicons were pooled in an equimolar ratio and sent to Majorbio Bio‐Pharm Technology Co. Ltd. (Shanghai, China) for paired‐end sequencing (2 × 300 bp) on an Illumina MiSeq platform. Raw reads were demultiplexed and quality‐processed using Trimmomatic and FLASH v1.2.7 according to our previous report (Min et al. 2025). Sequence denoising into ASVs was completed with the DADA2 plugin (Callahan et al. 2016) in QIIME 2 (Bolyen et al. 2019) in default settings. Taxonomic annotation of ASVs was performed using RDP Classifier v2.2 (confidence threshold = 0.7). All samples in this study were rarefied to 21,829 sequences, corresponding to an average Good's coverage of 99.80%.

### 2.6 Determination of Serum Parameters

Serum total protein (TP), albumin (ALB), total cholesterol (TC), triglycerides (TG), high‐density lipoprotein cholesterol (HDL), low‐density lipoprotein cholesterol (LDL), creatinine (CREA), urea (UREA), glucose (GLU), aspartate aminotransferase (AST), alanine aminotransferase (ALT), creatine kinase (CK), alkaline phosphatase (ALP), cholinesterase (CHE), γ‐glutamyl transferase (γ‐GT), immunoglobulin A (IgA), immunoglobulin G (IgG), immunoglobulin M (IgM), complement C3 (C3), complement C4 (C4), and sialic acid (SA) were quantified using a Mindray BS‐420 automatic biochemical analyzer (Mindray Bio‐Medical Electronics Co. Ltd., Shenzhen, China) with colorimetric kits (Beijing Zhongsheng Beikong Biological Technology Co. Ltd., Beijing, China) in accordance with the manufacturers' instructions.

Immunological parameters, including tumor necrosis factor‐α (TNF‐α), tumor necrosis factor‐β (TNF‐β), interferon‐γ (IFN‐γ), interleukin‐1β (IL‐1β), interleukin‐2 (IL‐2), IL‐4, IL‐6, IL‐8, IL‐10, IL‐17, IL‐18, and IL‐22 were determined using a Huawei Delang DR‐200BS microplate reader (Wuxi Huawei Delang Instrument Co. Ltd., Wuxi, China) with enzyme‐linked immunosorbent assay (ELISA) kits (Beijing Sino‐UK Institute of Biological Technology, Beijing, China) following the manufacturers' instructions.

Antioxidant and nitric oxide‐related parameters, including superoxide dismutase (SOD), glutathione peroxidase (GSH‐Px), glutathione (GSH), malondialdehyde (MDA), total antioxidant capacity (T‐AOC), nitric oxide (NO), hydrogen peroxide (H_2_O_2_), total nitric oxide synthase (T‐NOS), inducible nitric oxide synthase (iNOS), and constitutive nitric oxide synthase (cNOS) were measured using a Huawei Delang DR‐200BS microplate reader (Wuxi Huawei Delang Instrument Co. Ltd., Wuxi, China) with colorimetric kits (Beijing Sino‐UK Institute of Biological Technology, Beijing, China) according to the manufacturers' instructions.

### 2.7 Statistical Analysis

Bioinformatic processing of ruminal and fecal microbial datasets was performed on the Majorbio Cloud platform (http://cloud.majorbio.com/). After preliminary pre‐processing in Excel 2019, ASV‐level data were used to characterize microbial community composition and relative abundance. Alpha‐diversity metrics (Shannon, Simpson, Chao, Sobs) were computed using Mothur v1.30.2 (https://mothur.org/wiki/calculators/). PCoA based on Unweighted UniFrac distance combined with Adonis permutational test assessed inter‐sample microbial community dissimilarity. LEfSe with stringent all‐against‐all pairwise comparisons was executed on rarefied 16S rRNA reads, with an LDA score cutoff of 2.5.

To investigate functional associations of ruminal and fecal bacteria with ruminal fermentation, serum biochemical, immune and antioxidant indices, we built a Spearman's rank‐correlation matrix using R. This matrix comprised correlation coefficients between the top 10 significantly differential genera and target variables; only relationships with *p* < 0.05 and |*ρ*| ≥ 0.6 were retained.

The Shapiro–Wilk test (PROC UNIVARIATE, SAS v9.2; SAS Institute Inc., Cary, NC, USA) was adopted to check normality for growth performance, ruminal fermentation and serum biochemical datasets, while the standard error of the mean (SEM) was computed. Inter‐group comparisons were made using SPSS 18.0. Homogeneity of variance was examined before analysis. One‐way ANOVA was used to assess overall differences among groups; Duncan's multiple range test was adopted for post hoc multiple comparisons when variance homogeneity was satisfied. For data with heterogeneous variances, Welch's ANOVA was applied for overall significance testing, followed by the Games‐Howell test for pairwise comparisons. All data were shown as means, and *p* < 0.05 indicated statistical significance.

## 3 Results

### 3.1 Effects on Growth Performance in Sunit Sheep

The effects of supplementation strategies on the growth performance of Sunit sheep are presented in Table 1. Neither strategy significantly affected ADG (*p* > 0.05), nor did initial or final body weights differ among groups (*p* > 0.05).

**TABLE 1:** Effects of uncoated mannitol and coated mannitol on growth performance in three treatment groups.

Items	Treatment			SEM	*p*
	CON	UM	CM		
Initial body weight, kg	31.52	29.97	28.45	4.755	0.665
Final body weight, kg	33.65	34.05	31.43	5.394	0.762
ADG, g/d	152.68	291.52	212.95	38.626	0.063

*Note:* Results are presented as the mean and standard error of the mean (SEM), *n* = 8.

Abbreviations: ADG, Average Daily Gain; CM, Coated Mannitol group; CON, Control group; UM, Uncoated Mannitol group.

### 3.2 Effects on Rumen Fermentation Parameters in Sunit Sheep

Table 2 presents the effects of supplementation strategies on rumen fermentation in Sunit sheep. Compared with the CON group, both UM and CM groups showed a significant increase in rumen pH (*p* < 0.05), whereas concentrations of NH_3_–N, acetate, propionate, butyrate, and total SCFAs were significantly lower (*p* < 0.05). No significant differences in these parameters were observed between UM and CM groups (*p* > 0.05).

**TABLE 2:** Effects of uncoated mannitol and coated mannitol on rumen fermentation profiles in three treatment groups.

Items	Treatment			SEM	*p*
	CON	UM	CM		
pH	6.23^b^	6.90^a^	6.93^a^	0.072	< 0.001
NH_3_–N, mg/100 ml	16.37^a^	4.04^b^	3.21^b^	1.122	< 0.001
Total SCFAs, mmol/L	124.00^a^	68.65^b^	63.52^b^	5.692	< 0.001
Acetic acid, mmol/L	78.87^a^	47.42^b^	43.47^b^	3.121	< 0.001
Propionic acid, mmol/L	31.85^a^	12.90^b^	13.73^b^	2.229	< 0.001
Butyric acid, mmol/L	13.28^a^	8.33^b^	6.33^b^	1.027	< 0.001

*Note:* Results are presented as the mean and standard error of the mean (SEM), *n* = 8. ^a,b^Values within the same row that share different lowercase superscripts indicate significant differences (*p* < 0.05).

Abbreviations: CM, Coated Mannitol group; CON, Control group; Total SCFAs, total short‐chain fatty acids; UM, Uncoated Mannitol group.

### 3.3 Effects on the Microbiota Diversity of the Rumen and Feces in Sunit Sheep

As shown in Figures 1A–D and 2A–D, α‐diversity analysis revealed that the UM group did not differ significantly from the CON group in rumen or fecal microbial richness and diversity (*p* > 0.05). In the CM group, only the rumen microbial Sobs index was significantly different from the CON group (*p* < 0.05); no significant differences were detected in other diversity indices, and fecal microbial richness and diversity also showed no significant differences (*p* > 0.05).

**FIGURE 1:**
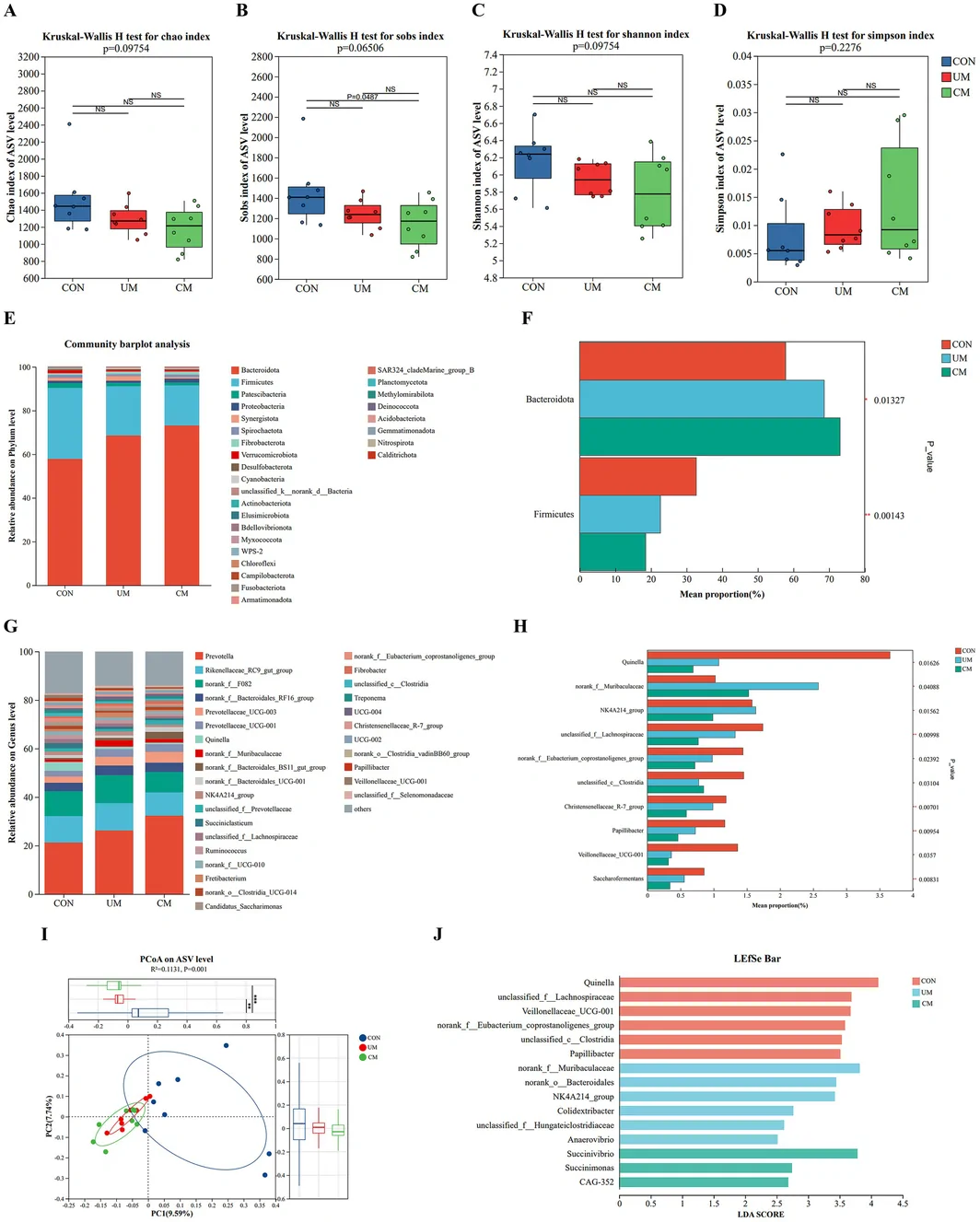
Effects of uncoated and coated mannitol on rumen microbes in sheep. The α‐diversity indices of rumen microbial communities in sheep among different treatment groups, with (A) Chao Index, (B) Sobs Index, (C) Shannon Index, and (D) Simpson Index. Rumen microbiota composition at the (E) phylum and (G) genus levels. Differences in rumen bacterial taxa of sheep at the (F) phylum and (H) genus levels; Alpha‐diversity indices were compared using the Kruskal–Wallis test, and corresponding *p*‐values are shown in each panel. (I) PCoA plot of rumen microbial communities at the ASV level. (J) The bacterial biomarkers in the rumen of sheep from the UM and CM were identified separately via LEfSe analysis, with a LDA score > 2.5. CON, Control group; CM, Coated Mannitol group; UM, Uncoated Mannitol group. *p* < 0.05 denotes a significant difference.

**FIGURE 2:**
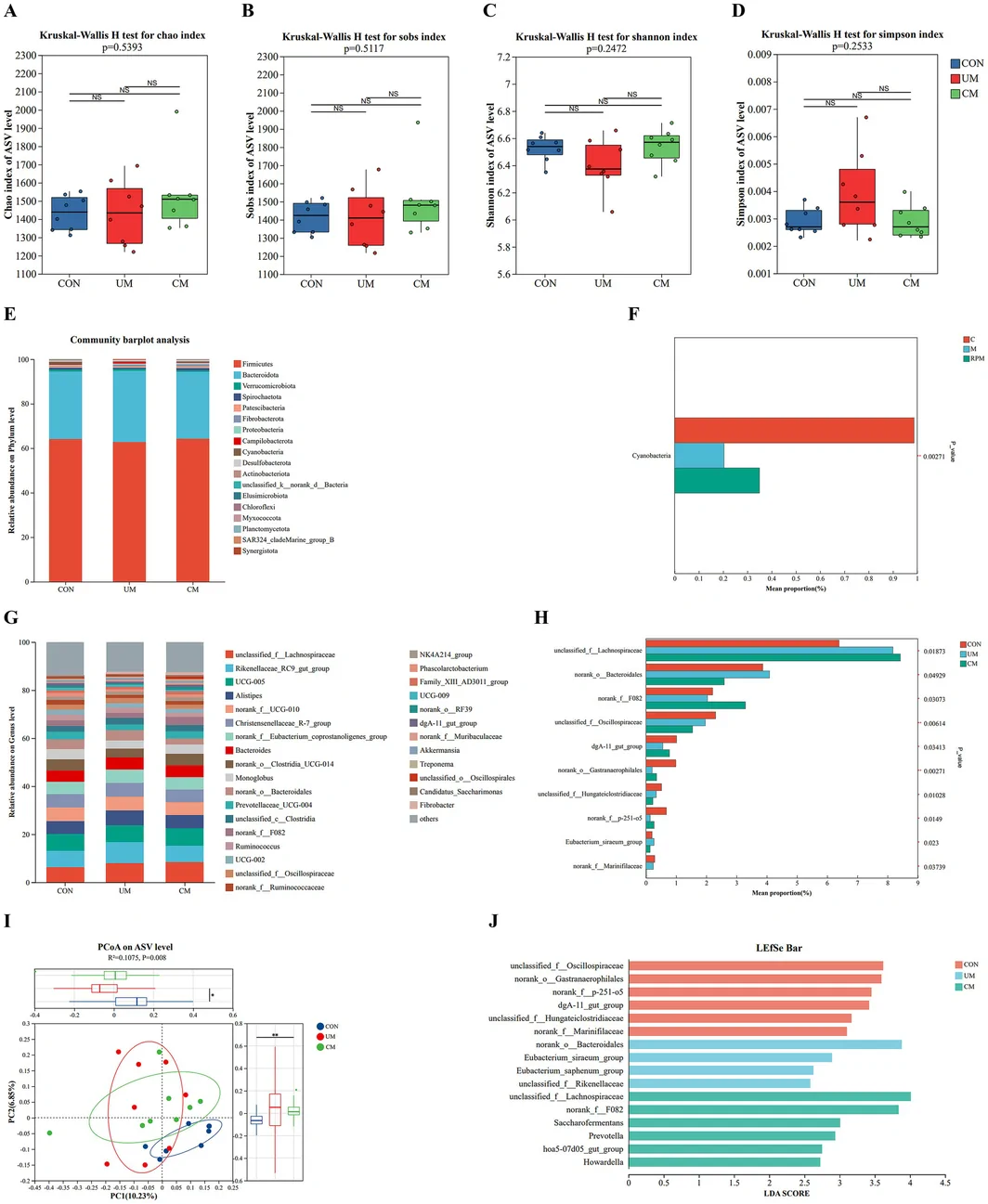
Effects of uncoated and coated mannitol on fecal microbes in sheep. The α‐diversity indices of fecal microbial communities in sheep among different treatment groups, with (A) Chao Index, (B) Sobs Index, (C) Shannon Index, and (D) Simpson Index. Fecal microbiota composition at the (E) phylum and (G) genus levels. Differences in fecal bacterial taxa of sheep at the (F) phylum and (H) genus levels; Alpha‐diversity indices were compared using the Kruskal–Wallis test, and corresponding *p*‐values are shown in each panel. (I) PCoA plot of fecal microbial communities at the ASV level. (J) The bacterial biomarkers in the fecal of sheep from the UM and CM were identified separately via LEfSe analysis, with a LDA score > 2.5. CON, Control group; CM, Coated Mannitol group; UM, Uncoated Mannitol group. *p* < 0.05 denotes a significant difference.

As shown in Figure 1I, principal coordinate analysis (PCoA) based on unweighted UniFrac distance revealed that the rumen microbial community in the treatment groups was clearly separated from the CON group. For the fecal microbial community (Figure 2I), both UM and CM groups significantly altered the fecal microbial community structure.

### 3.4 Effects on the Rumen Microbial Community in Sunit Sheep

A total of 28 bacterial phyla and 362 genera were identified across all samples. Rumen microbial composition at the phylum level is shown in Figure 1E. Bacteroidota was the most dominant phylum in all groups, followed by Firmicutes, while other phyla (e.g., *Patescibacteria*) exhibited low abundances. At the genus level (Figure 1G), *Prevotella* was the most abundant, followed by *Rikenellaceae_RC9_gut_group*, *Quinella*, *norank_f__Bacteroidales_RF16_group*, and *norank_f__F082*.

The Kruskal–Wallis *H* test showed that at the phylum level (Figure 1F), both UM and CM groups had significantly higher Bacteroidota and lower Firmicutes abundances than the CON group (*p* < 0.05). At the genus level (Figure 1H), *Quinella*, *norank_f__Muribaculaceae*, *Veillonellaceae_UCG‐001*, and *unclassified_c__Clostridia* varied numerically but not significantly (*p* > 0.05). The CM group had significantly lower abundances of *NK4A214_group*, *norank_f__Eubacterium_coprostanoligenes_group*, *Christensenellaceae_R‐7_group*, *Papillibacter*, *Saccharofermentans*, and *unclassified_f__Lachnospiraceae* than the CON group (*p* < 0.05). No significant differences in these taxa were observed between UM and CM groups (*p* > 0.05).

LEfSe analysis was used to identify differential taxa (Figure 1J). The CON group was enriched in *Quinella*, *unclassified_f__Lachnospiraceae*, *Papillibacter*, *Veillonellaceae_UCG‐001*, *norank_f__Eubacterium_coprostanoligenes_group*, and *unclassified_c__Clostridia*. The UM group was characterized by *norank_f__Muribaculaceae*, *norank_o__Bacteroidales*, *Anaerovibrio*, *NK4A214_group*, *Colidextribacter*, and *unclassified_f__Hungateiclostridiaceae*. The CM group was enriched in *Succinivibrio*, *Succinimonas*, and *CAG‐352*.

### 3.5 Effects on the Fecal Microbial Community in Sunit Sheep

A total of 18 bacterial phyla and 312 genera were identified across all fecal samples. Fecal microbial composition at the phylum level is shown in Figure 2E. Firmicutes was the most dominant phylum in all groups, followed by Bacteroidota, while other phyla (e.g., Verrucomicrobiota, Spirochaetota) showed low abundances. At the genus level (Figure 2G), *unclassified_f__Lachnospiraceae* was the most abundant, followed by *Rikenellaceae_RC9_gut_group*, *UCG‐005*, *Alistipes*, and *norank_f__UCG‐010*.

Kruskal–Wallis *H* test revealed that at the phylum level (Figure 2F), both UM and CM groups had significantly lower Cyanobacteria abundance than the CON group (*p* < 0.05). At the genus level (Figure 2H), the UM group exhibited significantly lower abundances of *norank_o__Gastranaerophilales* and *norank_f__p‐251‐o5*, and higher *unclassified_f__Lachnospiraceae* than the CON group (*p* < 0.05). The CM group had significantly lower abundances of *norank_f__Marinifilaceae*, *unclassified_f__Oscillospiraceae*, and *unclassified_f__Hungateiclostridiaceae*, and higher *unclassified_f__Lachnospiraceae* compared with the CON group (*p* < 0.05). Other genera including *norank_o__Bacteroidales*, *norank_f__F082*, *dgA‐11_gut_group*, and *Eubacterium_siraeum_group* varied numerically but not significantly (*p* > 0.05).

LEfSe analysis identified differential fecal microbial taxa (Figure 2J). The CON group was enriched in *unclassified_f__Oscillospiraceae*, *norank_o__Gastranaerophilales*, *norank_f__p‐251‐o5*, *dgA‐11_gut_group*, *unclassified_f__Hungateiclostridiaceae*, and *norank_f__Marinifilaceae*. The UM group was characterized by *norank_o__Bacteroidales*, *Eubacterium_siraeum_group*, *Eubacterium_saphenum_group*, and *unclassified_f__Rikenellaceae*. The CM group was enriched in *unclassified_f__Lachnospiraceae*, *norank_f__F082*, *Saccharofermentans*, *Prevotella*, hoa5‐07d05_gut_group, and *Howardella*.

### 3.6 Effects on Serum Biochemical Indices in Sunit Sheep

Table 3 presents the effects of supplementation strategies on serum biochemical indices of Sunit sheep. Significant differences were observed among groups: Compared with the CON group, the UM group had significantly higher TP, ALB, CREA and AST, but lower UREA levels (*p* < 0.05); TC, TG, HDL, LDL, GLU, ALP, CHE, γ‐GT, SA, ALT and CK showed no significant changes (*p* > 0.05). Similarly, the CM group exhibited significantly increased TP, ALB, and AST, and decreased UREA (*p* < 0.05); Other indices including TC, TG, HDL, LDL, ALT, CK, ALP, CHE, γ‐GT, GLU, SA, and CREA did not differ significantly (*p* > 0.05).

**TABLE 3:** Effects of uncoated mannitol and coated mannitol on serum biochemical indices in three treatment groups.

Items	Treatment			SEM	*p*
	CON	UM	CM		
TP g/L	63.52^b^	68.97^a^	69.66^a^	1.262	0.004
ALB, g/L	29.58^b^	32.62^a^	32.59^a^	0.824	0.024
TC, mmol/L	1.39	1.62	1.66	0.105	0.178
TG, mmol/L	0.26	0.23	0.25	0.021	0.567
HDL, mmol/L	0.68	0.80	0.75	0.042	0.155
LDL, mmol/L	0.40	0.43	0.53	0.048	0.150
CREA, umol/L	92.16^b^	100.36^a^	98.15^ab^	2.269	0.049
UREA, mmol/L	9.73^a^	7.43^b^	7.62^b^	0.496	0.006
GLU, mmol/L	6.34	5.66	5.19	0.360	0.100
AST, U/L	116.10^b^	143.22^a^	140.80^a^	6.530	0.014
ALT, U/L	17.37	22.99	20.45	1.900	0.137
CK, U/L	250.29	386.36	252.27	43.172	0.057
ALP, U/L	258.75	391.82	271.13	28.678	0.056
CHE, U/L	2479.08	3263.24	2938.26	672.071	0.629
γ‐GT, U/L	54.95	57.76	53.45	2.802	0.552
SA, mg/dL	62.69	60.27	45.16	5.450	0.069

*Note:* Results are presented as the mean and standard error of the mean (SEM), *n* = 8. ^a,b^Values within the same row that share different lowercase superscripts indicate significant differences (*p* < 0.05).

Abbreviations: γ‐GT, γ‐glutamyl transferase; ALB, albumin; ALP, alkaline phosphatase; ALT, alanine aminotransferase; AST, aspartate aminotransferase; CHE, cholinesterase; CK, creatine kinase; CM, Coated Mannitol group; CON, Control group; CREA, creatinine; GLU, glucose; HDL, high‐density lipoprotein; LDL, low‐density lipoprotein; SA, serum sialic acid; TC, total cholesterol; TG, triglyceride; TP, total protein; UM, Uncoated Mannitol group; UREA, urea.

### 3.7 Effects on Immune Indices in Sunit Sheep

Table 4 summarizes the effects on serum immune indices in Sunit sheep. No significant differences in any immune indices were observed between the UM and CON groups (*p* > 0.05). Compared with the CON group, the CM group had significantly higher IL‐4, IgA, and C3 levels, but lower TNF‐β, IFN‐γ, and IL‐22 concentrations (*p* < 0.05), other immune indices showed no significant differences (*p* > 0.05). Compared with the CM group, the UM group had significantly higher TNF‐β, IFN‐γ, and IL‐22 levels, but lower IL‐4, IgA, and C3 levels (*p* < 0.05); no significant differences were detected in the other indices (*p* > 0.05).

**TABLE 4:** Effects of uncoated mannitol and coated mannitol on the serum immune indices in three treatment groups.

Items	Treatment			SEM	*p*
	CON	UM	CM		
TNFα, pg/mL	51.75	52.37	43.61	2.316	0.067
TNFβ, pg/mL	34.71^a^	34.17^a^	28.91^b^	1.677	0.043
IFN‐γ, pg/mL	39.44^a^	39.00^a^	34.26^b^	1.421	0.032
IL‐1β, pg/mL	22.70	23.14	18.13	1.531	0.057
IL‐2, pg/mL	223.04	226.58	192.21	9.829	0.102
IL‐4, pg/mL	4.66^b^	5.03^b^	5.91^a^	0.286	0.017
IL‐6, pg/mL	129.60	124.89	112.02	5.234	0.070
IL‐8, pg/mL	44.71	44.14	36.39	2.585	0.060
IL‐10, pg/mL	10.99	10.92	12.54	0.781	0.274
IL‐17, pg/mL	34.38	34.23	26.22	2.792	0.084
IL‐18, pg/mL	101.10	101.94	86.67	5.375	0.102
IL‐22, pg/mL	32.85^a^	31.73^a^	25.99^b^	1.762	0.026
IgA, g/L	1.09^b^	1.14^b^	1.52^a^	0.122	0.044
IgG, g/L	15.83	15.77	18.20	0.832	0.085
IgM, g/L	0.66	0.56	0.71	0.052	0.158
C3, g/L	0.48^b^	0.48^b^	0.61^a^	0.038	0.045
C4, g/L	0.13	0.13	0.17	0.014	0.061

*Note:* Results are presented as the mean and standard error of the mean (SEM), *n* = 8. ^a,b^Values within the same row that share different lowercase superscripts indicate significant differences (*p* < 0.05).

Abbreviations: C3, complement component 3; C4, complement component 4; CM: Coated Mannitol group; CON: Control group; IFN‐γ, interferon‐γ; IgA, immunoglobulin A; IgG, immunoglobulin G; IgM, immunoglobulin M; IL‐10, interleukin‐10; IL‐17, interleukin‐17; IL‐18, interleukin‐18; IL‐1β, interleukin‐1β; IL‐2, interleukin‐2; IL‐22, interleukin‐22; IL‐4, interleukin‐4; IL‐6, interleukin‐6; IL‐8, interleukin‐8; SEM, standard error of the mean; TNF‐α, tumor necrosis factor α; TNF‐β, tumor necrosis factor β; UM: Uncoated Mannitol group.

### 3.8 Effects on Antioxidant Indices in Sunit Sheep

Table 5 presents the effects on serum antioxidant indices in Sunit sheep. Compared to the CON group, the UM group had significantly higher iNOS activity and lower cNOS activity (*p* < 0.05), with no significant differences in other indices (*p* > 0.05). The CM group showed significantly higher SOD and GSH‐Px activities and lower MDA content than the CON group (*p* < 0.05), while other indices did not differ significantly (*p* > 0.05). Compared with the CM group, the UM group had significantly lower SOD, GSH‐Px, NO and cNOS, and higher MDA (*p* < 0.05); no significant differences were observed in the other indices (*p* > 0.05).

**TABLE 5:** Effects of uncoated mannitol and coated mannitol on the serum antioxidant indices in three treatment groups.

Items	Treatment			SEM	*p*
	CON	UM	CM		
SOD, U/mL	77.43^b^	76.47^b^	85.91^a^	2.614	0.035
GSH‐PX, U/mL	114.59^b^	111.14^b^	145.72^a^	8.150	0.012
GSH, umol/L	4.85	4.72	6.21	0.489	0.081
MDA, nmol/mL	4.16^a^	4.27^a^	3.05^b^	0.349	0.041
T‐AOC, U/mL	37.08	39.27	45.62	2.676	0.087
NO, umol/L	38.77^ab^	35.36^b^	45.35^a^	2.382	0.010
H_2_O_2_, mmol/L	60.44	60.08	44.10	6.183	0.127
T‐NOS, U/mL	19.81	19.31	22.99	1.157	0.140
iNOS, U/mL	9.72^b^	14.10^a^	11.38^ab^	1.084	0.002
cNOS, U/mL	10.09^a^	5.20^b^	11.61^a^	1.355	0.008

*Note:* Results are presented as the mean and standard error of the mean (SEM), *n* = 8. ^a,b^Values within the same row that share different lowercase superscripts indicate significant differences (*p* < 0.05).

Abbreviations: CM, Coated Mannitol group; cNOS, constitutive nitric oxide synthase; CON, Control group; GSH, glutathione; GSH‐PX, glutathione peroxidase; H_2_O_2_, hydrogen peroxide; iNOS, inducible nitric oxide synthase; MDA, malondialdehyde; NO, nitric oxide; SEM, Standard Error of the Mean; SOD, superoxide dismutase; T‐AOC, total antioxidant capacity; T‐NOS, total nitric oxide synthase; UM, Uncoated Mannitol group.

### 3.9 Correlation Between Rumen Bacteria and Rumen Fermentation, and Relationships Between Rumen or Fecal Bacteria and Serum Indices

The interrelationships among rumen fermentation parameters, serum indices, and differential bacterial taxa were analyzed (Figure 3). *Quinella* was strongly positively correlated with acetate (Ac), butyrate (Bu), and Total SCFAs (*ρ* = 0.677, *ρ* = 0.612, *ρ* = 0.657; all *p* < 0.001). *Unclassified_f__Lachnospiraceae* was significantly positively correlated with NH_3_–N (*ρ* = 0.714, *p* = 0.001). *Saccharofermentans* showed significant positive correlations with NH_3_–N, Ac, Bu, and Total SCFAs (*ρ* = 0.716, *ρ* = 0.643, *ρ* = 0.619, *ρ* = 0.641; *p* ≤ 0.001). *Papillibacter* was markedly positively correlated with Ac, propionate (Pr), Bu, and Total SCFAs (*ρ* = 0.641, *ρ* = 0.641, *ρ* = 0.836, *ρ* = 0.706; *p* ≤ 0.001). *NK4A214_group* was significantly positively correlated with Bu (*ρ* = 0.644, *p* = 0.001). *Norank_f__Eubacterium_coprostanoligenes_group* was significantly positively correlated with Ac, Total SCFAs, and IL‐6 (*ρ* = 0.609, *ρ* = 0.640, *ρ* = 0.648; *p* ≤ 0.002). *Christensenellaceae_R‐7_group* was strongly negatively correlated with IgG and IL‐4 (*ρ* = −0.655, *ρ* = −0.611; *p* ≤ 0.002).

**FIGURE 3:**
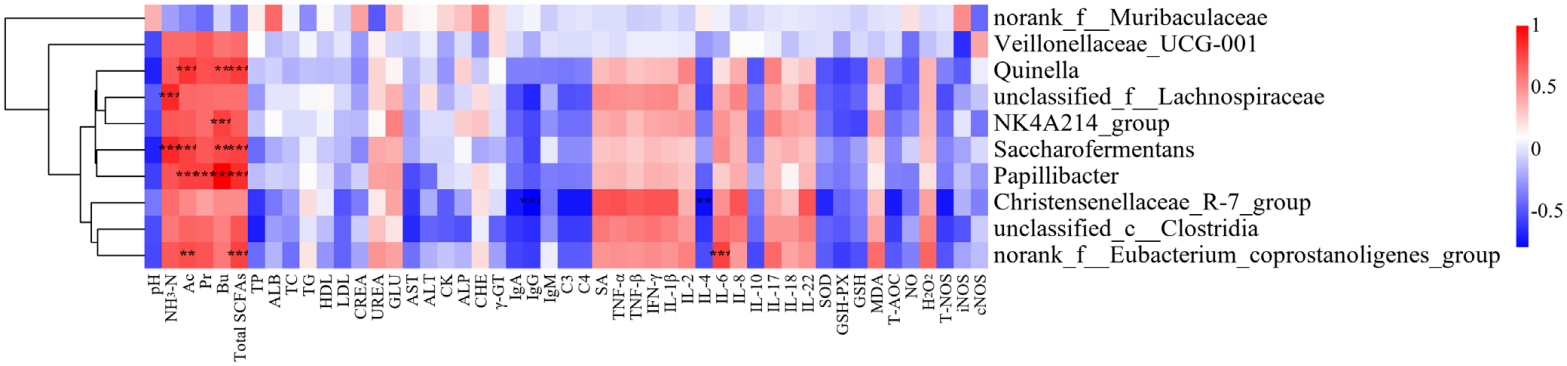
Correlations between the relative abundances of rumen microbiota and rumen fermentation indices, serum biochemical indices, immune indices, and antioxidant indices. Positive correlations are shown in red, while negative correlations are in blue. The color intensity of the blocks is proportional to the absolute value of the correlation coefficient (|*ρ*|) within the corresponding correlation group. *p < 0.05, **p < 0.01, ***p < 0.001; γ‐GT, γ‐glutamyl transferase; Ac, Acetic acid; ALB, albumin; ALP, alkaline phosphatase; ALT, alanine aminotransferase; AST, aspartate aminotransferase; Bu, Butyric acid; C3, complement component 3; C4, complement component 4; CHE, cholinesterase; CK, creatine kinase; cNOS, constitutive nitric oxide synthase.; CREA, creatinine; GLU, glucose; GSH, glutathione; GSH‐PX, glutathione peroxidase; H_2_O_2_, hydrogen peroxide; HDL, high‐density lipoprotein; IFN‐γ, interferon‐γ; IgA, immunoglobulin A; IgG, immunoglobulin G; IgM, immunoglobulin M; IL‐10, interleukin‐10; IL‐17, interleukin‐17; IL‐18, interleukin‐18; IL‐1β, interleukin‐1β; IL‐2, interleukin‐2; IL‐22, interleukin‐22; IL‐4, interleukin‐4; IL‐6, interleukin‐6; IL‐8, interleukin‐8; iNOS, inducible nitric oxide synthase; LDL, low‐density lipoprotein; MDA, malondialdehyde; NH_3_–N, Ammonia Nitrogen; NO, nitric oxide; Pr, Propionic acid; SA, serum sialic acid; SOD, superoxide dismutase; T‐AOC, total antioxidant capacity; TC, total cholesterol; TG, triglyceride; TNF‐α, tumor necrosis factor α; TNF‐β, tumor necrosis factor β; T‐NOS, total nitric oxide synthase; Total SCFAs, Total Short‐Chain Fatty Acids; TP, total protein; UREA, urea. This correlation analysis was exploratory. Raw *p*‐values are presented without adjustment for multiple comparisons; hence these associations should be interpreted with caution.

Similarly, associations between serum indices and differential fecal bacterial taxa were examined (Figure 4). *Norank_f__p‐251‐o5* was significantly negatively correlated with TC (*ρ* = −0.621, *p* = 0.001). *Norank_o__Gastranaerophilales* was significantly negatively correlated with TP (*ρ* = −0.652, *p* = 0.001) but positively correlated with UREA (*ρ* = 0.611, *p* = 0.002).

**FIGURE 4:**
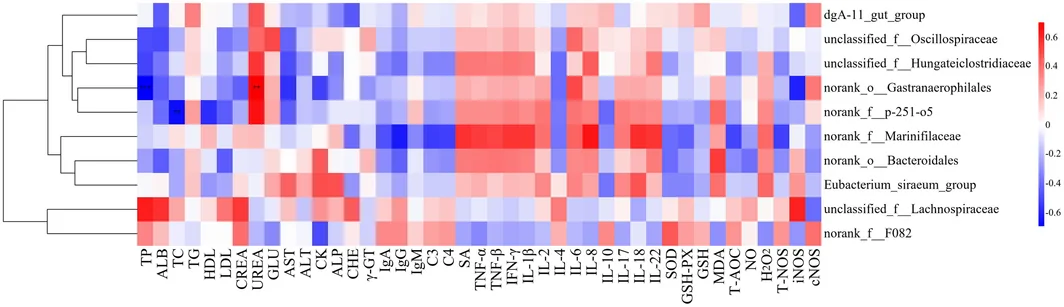
Correlations between the relative abundances of fecal microbiota and serum biochemical indices, immune indices, and antioxidant indices. Positive correlations are shown in red, while negative correlations are in blue. The color intensity of the blocks is proportional to the absolute value of the correlation coefficient (|*ρ*|) for the corresponding correlation group. *p < 0.05, **p < 0.01, ***p < 0.001. γ‐GT, γ‐glutamyl transferase; ALB, albumin; ALP, alkaline phosphatase; ALT, alanine aminotransferase; AST, aspartate aminotransferase; C3, complement component 3; C4, complement component 4; CHE, cholinesterase; CK, creatine kinase; cNOS, constitutive nitric oxide synthase.; CREA, creatinine; GLU, glucose; GSH, glutathione; GSH‐PX, glutathione peroxidase; H_2_O_2_, hydrogen peroxide; HDL, high‐density lipoprotein; IFN‐γ, interferon‐γ; IgA, immunoglobulin A; IgG, immunoglobulin G; IgM, immunoglobulin M; IL‐10, interleukin‐10; IL‐17, interleukin‐17; IL‐18, interleukin‐18; IL‐1β, interleukin‐1β; IL‐2, interleukin‐2; IL‐22, interleukin‐22; IL‐4, interleukin‐4; IL‐6, interleukin‐6; IL‐8, interleukin‐8; iNOS, inducible nitric oxide synthase; LDL, low‐density lipoprotein; MDA, malondialdehyde; NO, nitric oxide; SA, serum sialic acid; SOD, superoxide dismutase; T‐AOC, total antioxidant capacity; TC, total cholesterol; TG, triglyceride; TNF‐α, tumor necrosis factor α; TNF‐β, tumor necrosis factor β; T‐NOS, total nitric oxide synthase; TP, total protein; UREA, urea. This correlation analysis was exploratory. Raw *p*‐values are presented without adjustment for multiple comparisons; hence these associations should be interpreted with caution.

## 4 Discussion

### 4.1 Effects of Uncoated and Coated Mannitol on Growth Performance of Sunit Sheep

Studies in hindgut‐fermenting monogastric rabbits have shown that dietary D‐mannitol serves as an energy substrate for cecal microbes, enhances fiber digestibility and modulates cecotrophy‐mediated nutrient recycling. Under specific experimental conditions, these beneficial responses can improve rabbit daily weight gain and feed efficiency (Hanieh and Sakaguchi 2009; Li et al. 2012). However, relevant studies in ruminants remain limited. Previous studies reported that mannitol supplementation in grazing Mongolian sheep (Min et al. 2025), and rumen‐protected glucose and taurine in yaks, had no significant effects on growth performance (Chen et al. 2025). Consistent with these, our study found no significant ADG differences among Sunit sheep in UM or CM group, possibly due to low mannitol dose and short feeding duration. Collectively, dietary mannitol can regulate rumen microbial community and fermentation characteristics, but these effects are insufficient to improve growth performance in sheep.

### 4.2 Effects of Uncoated and Coated Mannitol on Rumen Fermentation Parameters in Sunit Sheep

Rumen fermentation stability is fundamental to ruminants. Prebiotics can modulate rumen‐fermentation‐related parameters (Klassen et al. 2023), with similar regulatory effects of rumen‐protected additives observed in yaks (Wang et al. 2024). In our study, both UM and CM groups significantly increased ruminal pH to approximately 6.9 and reduced total ruminal SCFAs, acetate, propionate, and butyrate. Notably, some ruminant‐based studies have reported that certain functional oligosaccharides can increase ruminal SCFAs concentrations, although inconsistent results have also been observed, which is in contrast to our findings (Chang et al. 2022; Wang et al. 2021; Zheng, Zhang, et al. 2021; Zheng, Zhou, et al. 2021) contradicting our findings. This discrepancy may stem from two factors: First, mannitol modulates rumen microbiota by suppressing acidogenic bacteria and promoting non‐acidogenic beneficial bacteria, stabilizing the ruminal microenvironment and improving nutrient digestion; Second, rumen‐protected coating cannot achieve complete protection, partially uncoated or prematurely released mannitol enters the rumen, exerting effects similar to uncoated mannitol and regulating key fermentation parameters.

Previous studies confirm *Papillibacter* directly synthesizes acetate and butyrate (Liu et al. 2021; Xue et al. 2020), and its positive correlation with propionate may reflect indirect regulation of propionate‐producing bacteria. *Saccharofermentans* plays a key role in carbohydrate fermentation, producing lactate, acetate, and butyrate (Ahmad et al. 2022; Li, Li, et al. 2024). *Quinella* produces lactate and propionate as its major fermentation end products (Kumar et al. 2022). *Eubacterium strain HL* (a core representative of *norank_f__Eubacterium_coprostanoligenes_group*) produces acetate, with succinate and formate as fermentation byproducts (Freier et al. 1994). Additionally, *NK4A214_group* (a typical butyrate‐producing taxon) shows a significant positive correlation between its abundance and butyrate production (Li et al. 2023). Notably, these microorganisms' abundances decreased significantly only in the CM group, accompanied by reduced total SCFAs. This suggests uncoated mannitol is rapidly degraded in the rumen (short regulatory duration, mild inhibition of target bacteria), while coated mannitol (sustained release) exerts a more persistent regulatory effect.

Both mannitol treatments significantly reduced ruminal NH_3_–N concentrations, consistent with previous reports that chicory inulin, galactooligosaccharides (GOS), chitosan, and rumen‐protected folic acid (RPFA) exert similar effects in ruminants (Bett Kipkorir et al. 2024; Pen et al. 2007; Goiri et al. 2010; Wang et al. 2020). Uncoated and coated mannitol likely act via the same microbial pathway: enhancing rumen microbes' capture and utilization of NH_3_–N (e.g., accelerating microbial protein synthesis) to reduce accumulation and improve fermentation efficiency. In this study, ruminal NH_3_–N decreased by 75%–80% in UM and CM groups. This large relative reduction arises partly from the relatively high baseline NH_3_–N concentration in the CON group, yet the final concentrations of 3–4 mg/100 mL remain physiologically relevant. Although this mechanism was originally described in rabbit caecum, mannitol may similarly shunt blood urea‐nitrogen toward microbial nitrogen synthesis within the microbially dense rumen (Xiao et al. 2012). Concurrent with the substantial decline in ruminal NH_3_–N, mannitol supplementation significantly reduced total and individual volatile fatty acid concentrations. This fermentative profile differs from conventional prebiotics, which commonly promotes carbohydrate fermentation and boosts SCFA production. Variations in animal breed, basal diet and sampling timing may account for the inconsistencies between the present results and previous literature. Furthermore, *unclassified_f__Lachnospiraceae* and *Saccharofermentans* abundances were significantly positively correlated with ruminal NH_3_–N, and both declined markedly only in the CM group. These two taxa may serve as potential biomarkers for ruminal nitrogen metabolism health, though further validation is needed.

Min et al. (2025) found no ruminal fermentation changes with 10 g/kg mannitol in grazing Mongolian sheep. Conversely, 2 g/kg uncoated or coated mannitol elevated ruminal pH and lowered ammonia‐nitrogen and short‐chain fatty acids in indoor‐raised Sunit sheep. Such discrepancies may arise from variations in breed, feeding regime, and mannitol inclusion level. The 2 g/kg dose appeared to exert stronger rumen‐modulating effects. Collectively, both treatments modulated ruminal fermentation, and coated mannitol had relatively superior performance.

### 4.3 Effects of Uncoated and Coated Mannitol on the Rumen Microbial Community of Sunit Sheep

To investigate the effects of different mannitol preparations on rumen microbiota in sheep, this study systematically compared rumen microbial community characteristics between UM and CM groups. Results showed that Bacteroidetes and Firmicutes were the dominant phyla in the rumen of Sunit sheep, consistent with previous studies in sheep, goats (Min et al. 2024), and lambs (Wang, Wu, et al. 2025). Patescibacteria and Proteobacteria were the next most abundant, in line with findings by Wang, Wu, et al. (2025) and Du et al. (2025) in Inner Mongolia cashmere goats.

Compared with the CON group, both treatment groups significantly altered rumen microbial communities. At the phylum level, *Bacteroidota* abundance increased while Firmicutes decreased, consistent with the regulatory effects of mannan‐rich fraction (MRF) on lamb rumen microbiota (Hu et al. 2024). Bacteroidota encodes abundant oligosaccharide‐degrading genes and mediates polysaccharide catabolism and amino acid utilization, whereas Firmicutes mainly degrades cellulose‐based carbohydrates (Zhao et al. 2024). We hypothesize that mannitol remodels the rumen microbial community to favor simple oligosaccharide utilizaiton by altering carbohydrate metabolic competition, though the underlying mechanism remains unclear. Excessive *Quinella* impairs ruminal carbohydrate utilization (Kumar et al. 2022); although *Quinella* only showed a numerical reduction in the present study, this trend may alleviate such interference and improve the nutrient conversion efficiency. Both treatments also exhibited a numerical increasing trend in *norank_f__Muribaculaceae*, a family that degrades various polysaccharides (Ormerod et al. 2016). *Christensenellaceae_R‐7_group* was significantly reduced only in the CM group; this genus contributes to immune regulation, gastrointestinal homeostasis, and structural carbohydrate degradation (Beaumont et al. 2017; Zhang et al. 2025), so its reduction may affect host immunity, microbial balance, and nutrient digestion. In conclusion, mannitol intervention effectively optimizes rumen fermentation in sheep by improving carbohydrate utilization and enhancing polysaccharide degradation capacity.

### 4.4 Identification of Biomarker Rumen Microorganisms Regulated by Uncoated and Coated Mannitol via Linear Discriminant Analysis (LDA)

A LEfSe analysis (LDA score threshold = 2.5) was used to compare rumen bacterial taxa among CON, UM, and CM groups. Compared with the CON group, the UM group had higher abundances of *norank_f__Muribaculaceae*, *norank_o__Bacteroidales*, *NK4A214_group*, *Colidextribacter*, *unclassified_f__Hungateiclostridiaceae*, and *Anaerovibrio*. These taxa have well‐documented functions: *norank_f__Muribaculaceae* regulates lipid metabolism and polysaccharide degradation (Wu et al. 2023); *norank_o__Bacteroidales* modulates nitrogen metabolism (Li et al. 2025); *NK4A214_group* promotes fiber fermentation and SCFA production (Tang et al. 2024); *Colidextribacter* is an anti‐inflammatory probiotic (Zhang, Jiang, et al. 2023); *unclassified_f__Hungateiclostridiaceae* is hypothesized to have carbohydrate metabolic potential (Zhang, Jiang, et al. 2023); and *Anaerovibrio* mediates lipid metabolism (Liu et al. 2017). These results indicate that mannitol modulates rumen function by improving lipid, carbohydrate, and nitrogen metabolism, enhancing rumen‐host crosstalk, and stabilizing fermentation.

Compared with the CON group, the CM group had higher abundances of *Succinivibrio*, *Succinimonas*, and *CAG‐352*. Oligosaccharides can increase *Succinivibrio*, a polysaccharide‐degrading genus (Yu et al. 2017). *Succinimonas* uses carbohydrates to produce succinic and organic acids (Xu et al. 2025), suggesting that coated mannitol may provide substrates for its growth. *CAG‐352* is also increased by soybean isoflavones (SIFs) and improves rumen microecology (Shao et al. 2024). In summary, coated mannitol promotes polysaccharide‐degrading bacteria, provides fermentation substrates, and improves rumen metabolism.

### 4.5 Effects of Uncoated and Coated Mannitol on the Fecal Microbial Community of Sunit Sheep

This study showed that neither mannitol regimen significantly affected fecal microbial α‐diversity in Sunit sheep. However, PCoA revealed that treatments significantly altered fecal microbial community structure, with distinct remodeling direction between UM and CM groups.

Firmicutes and Bacteroidetes were the dominant fecal phyla, consistent with previous studies on goats (Lei et al. 2018) and dairy cows (Xin et al. 2019), though their abundances were unaffected. At the genus level, *unclassified_f__Lachnospiraceae* and *unclassified_f__Oscillospiraceae* differed significantly between groups. For *norank_o__Bacteroidales*, *norank_f__F082*, and *dgA‐11_gut_group*, only numerical trends were observed: UM tended to have higher *norank_o__Bacteroidales* but lower *norank_f__F082* and *dgA‐11_gut_group*, while CM displayed reduced *norank_o__Bacteroidales* and *dgA‐11_gut_group* and increased *norank_f__F082*. Research on *norank_o__Bacteroidales* is limited; only studies on Xinjiang fine‐wool sheep confirm its role in ruminal amino acid metabolism (Li et al. 2025), so its relatively higher abundance in the UM group may relate to host nitrogen metabolism regulation. *Unclassified_f__Oscillospiraceae* is negatively correlated with serum TC/TG (Zhu and Hou 2024) and has fiber‐degrading capabilities (Firrincieli et al. 2024), so its reduction may affect lipid metabolism and fiber digestion. *Norank_f__F082* promotes non‐structural carbohydrate utilization (Zhang, Huang, et al. 2023) and correlates with amino acid biosynthesis (Guo et al. 2024), so this numerical trend in its abundance may affect fiber degradation. D*gA‐11_gut_group* is associated with lipid metabolism disorders (Zhang et al. 2021), and this numerical decline in its abundance suggests that mannitol may alleviate lipid imbalance by potentially restraining its proliferation.

In summary, mannitol effectively reshapes the intestinal microbial community structure of sheep, with distinct regulatory effects between regimens. UM may enhance nitrogen utilization given by the relatively higher abundance of *norank_o__Bacteroidales*; CM may strengthen carbohydrate metabolism given by the relatively elevated *norank_f__F082* and regulate lipid metabolism and fiber digestion through the statistically significant reduction of *unclassified_f__Oscillospiraceae*. Both treatments tend to alleviate lipid disorders, accompanied by the relatively lower abundance of *dgA‐11_gut_group*.

### 4.6 Identification of Biomarker Fecal Microorganisms Regulated by Uncoated and Coated Mannitol via LDA

The LEfSe method was used to compare bacterial taxa among the three sheep groups. Results showed significantly higher abundances of *norank_o__Bacteroidales*, *Eubacterium_siraeum_group*, *Eubacterium_saphenum_group*, *unclassified_f__Rikenellaceae*, and *Lachnospiraceae_NC2004_group* in the UM group.

Previous studies confirmed that multiple *Eubacterium* strains synthesize butyrate (critical for intestinal physiology and microecological homeostasis) (Mukherjee et al. 2020). Adequate *Rikenellaceae* reduces colitis risk and stabilizes the microbial community (Palacios‐González et al. 2019), while *Lachnospiraceae* degrades indigestible plant components into SCFAs to promote intestinal health (Biddle et al. 2013). Thus, uncoated mannitol may stimulate beneficial gut bacteria proliferation and metabolism, supporting intestinal microecological homeostasis and gut health.

In the CM group, *unclassified_f__Lachnospiraceae, a uric acid‐degrading bacterial taxon was enriched* (Wu et al. 2025); *norank_f__F082*, *Saccharofermentans*, *Prevotella*, *hoa5‐07d05_gut_group*, and *Howardella* were significantly more abundant. *Saccharofermentans* (phylum Firmicutes) is negatively correlated with intestinal inflammation‐related gene expression but positively correlated with ZO‐1 (Kim et al. 2024), potentially alleviating intestinal dysbiosis. *Prevotella* degrades various polysaccharides (Precup and Vodnar 2019), and *Howardella* decomposes urea to produce ATP (Liu et al. 2024). Overall, coated mannitol may selectively regulate functional gut microbiota, optimizing intestinal metabolism, inhibiting inflammation, enhancing intestinal barrier function, and improving host health and nutrient utilization.

### 4.7 Effects of Uncoated and Coated Mannitol on Serum Biochemical Parameters

Elevated TP and ALB levels reflect improved protein nutritional status, enhanced nitrogen utilization and superior hepatic synthetic capacity in ruminants (Abebe et al. 2025; Arfuso et al. 2024). In this experiment, serum TP and ALB were significantly increased in the UM group, consistent with dietary MOS supplementation in Dezhou donkeys (Wang, Wang, et al. 2025). Dietary composition modification can induce adaptive changes in hepatic metabolic biomarkers. Ma et al. (2024) reported that partial replacement of the basal diet with hydroponic barley seedlings increased serum AST and ALT activities in lactating ewes; this response was interpreted as the enhanced hepatic metabolic activity rather than hepatocellular damage, accompanied by improved protein nutritional status and alleviated oxidative stress. Uncoated mannitol did not alter lipid and glucose metabolism parameters (GLU, TC, TG), in line with previous results on wheat bran‐derived feruloyl oligosaccharide supplementation in lambs (Wang et al. 2019). Our study revealed a significant negative correlation between serum TP and the relative abundance of fecal *norank_o__Gastranaerophilales*. UM treatment markedly reduced the abundance of this taxon. These findings suggest that UM may help maintain serum TP levels, partly by modulating this microorganism, which may exert adverse effects on host protein metabolism. Previous studies have also linked this taxon to inflammatory responses and reduced growth performance (Xie et al. 2022). Optimized intestinal microbiota reduce nutrient antagonism and harmful metabolite to enhance protein synthesis efficiency, while regulated ruminal microbiota promote nutrient fermentative conversion, consistent with the reduced ruminal NH_3_–N level.

Serum UREA reflects body protein metabolism and amino acid balance; decreased levels indicate accelerated protein synthesis/utilization and improved amino acid efficiency (Beatson et al. 2019; Li, Titgemeyer, and Hanigan 2019). Serum UREA was significantly reduced in both experimental groups, consistent with Li et al. (2012), confirming mannitol's positive role in nutrient utilization. Notably, serum UREA was positively correlated with fecal *norank_o__Gastranaerophilales* abundance, implying this taxon may participate in amino acid metabolism under UM intervention (to be validated by follow‐up experiments). CREA showed only a marginal overall treatment effect. Numerically higher CREA values were observed in the UM group. CM exhibited intermediate creatinine concentrations, which overlapped statistically with both CON and UM. Taken together, both mannitol treatments altered sheep nutrient‐related serum profiles, yet robust evidence for renal‐protective benefits in the CM group is lacking.

### 4.8 Effects of Uncoated and Coated Mannitol on Immune Indicators

As a carbohydrate‐derived prebiotic, mannitol is easily degraded by ruminal microorganisms. Uncoated mannitol had no significant effect on sheep immune indicators, likely due to its low dose: only trace amounts crossed the rumen barrier to reach the hindgut, insufficient to alter dominant hindgut microbial composition and immune parameters. In contrast, coated mannitol resists rapid ruminal degradation; most reaches the hindgut (despite partial coating damage) to exert biological effects. Our results showed that coated mannitol significantly increased serum IL‐4, IgA, and C3, while reducing TNF‐β, IFN‐γ, and IL‐22, an immunomodulatory effect consistent with previous studies (Zhou et al. 2025; Deng et al. 2007). Correlation analysis revealed a significant negative correlation between IL‐4 and ruminal *Christensenellaceae_R‐7_group* abundance. As a key anti‐inflammatory cytokine, IL‐4 is functionally associated with this microbe, which participates in ruminant immune regulation, health homeostasis, and nutrient metabolism (Liu et al. 2025; Wei et al. 2024). Thus, coated mannitol‐induced changes in *Christensenellaceae_R‐7_group* may indirectly enhance host immunity via regulating IL‐4, though the specific mechanism requires further validation.

In conclusion, rumen‐degradation‐resistant coated mannitol effectively improves sheep immune status by downregulating pro‐inflammatory cytokines (e.g., TNF‐β, IFN‐γ) to reduce inflammatory damage and increasing immunoactive substances (e.g., IgA, C3, IL‐4) to enhance pathogen resistance, supporting animal immune health.

### 4.9 Effects of Uncoated and Coated Mannitol on Antioxidant Indicators

SOD, GSH‐Px activities, and MDA content are key indicators of organismal antioxidant capacity. Previous studies confirm that increased endogenous antioxidant enzymes (SOD, catalase (CAT), GSH‐Px) activities and reduced MDA (a lipid peroxidation product) levels reflect enhanced free radical scavenging and anti‐oxidative damage capacity (Cidan et al. 2025; Zheng, Zhang, et al. 2021; Zheng, Zhou, et al. 2021).

Polysaccharide‐based functional substances enhance antioxidant capacity (Wan et al. 2017; Li, Tong, et al. 2024). In this study, coated mannitol significantly increased sheep serum SOD and GSH‐Px activities and decreased MDA levels, indicating effective modulation of antioxidant function. In contrast, uncoated mannitol significantly increased iNOS and decreased cNOS activities, with no positive effect on antioxidant capacity. This is likely due to low effective dosage: uncoated mannitol was extensively degraded by rumen microorganisms, limiting hindgut delivery of active ingredients. Alternative explanations (e.g., rumen metabolic variation, mannitol target limitations) require further validation via dose‐gradient experiments and quantitative rumen degradation assessments.

## 5 Conclusions

In conclusion, under the experimental conditions of this study, dietary supplementation with either uncoated or coated mannitol had no significant effect on sheep growth performance. Both mannitol treatments appeared to optimize host nutrient metabolism, possibly via suppressing potentially pathogenic bacteria and modulating ruminal and intestinal microbiota. Furthermore, functional differences were observed between the two formulations. For immune function and antioxidant capacity, low‐dose coated mannitol (2 g/kg) exhibited favorable bioactivity, whereas equal‐dose uncoated mannitol exerted limited effects. This finding contrasts with our previous work, in which a higher dose (10 g/kg) of uncoated mannitol was required to achieve immune‐antioxidant benefits in Mongolian sheep. Accordingly, coated mannitol shows promising potential for enhancing immune and antioxidant capacities in sheep. Collectively, these results suggest that coating treatment may improve the biological performance of mannitol as a feed additive for sheep. Nevertheless, the present study has several inherent limitations. The relatively small number of experimental animals per group may weaken the statistical reliability, with the single dosage setting and short‐term feeding duration failing to clarify the dose‐dependent effects and long‐term regulatory mechanism for mannitol. In addition, only male sheep are used in this trial, which limits the general applicability of the results. Further systematic trials with multi‐dose gradients, longer feeding cycles and both male and female sheep are currently underway in our laboratory to consolidate and extend the current findings.

## Author Contributions

**Min Li:** data curation, formal analysis, investigation, software, methodology, visualization, writing – original draft. **Li Tao:** data curation, formal analysis, investigation, software. **Yang Li:** data curation, formal analysis, investigation, software. **Chao Wang:** investigation, supervision, writing – review and editing. **Fugui Yu:** investigation, supervision, writing – review and editing. **Jian Gong:** investigation, supervision, writing – review and editing. **Min Xiao:** conceptualization, funding acquisition, methodology, project administration, supervision, writing – review and editing.

## Funding

This work was supported by Natural Science Foundation of Inner Mongolia Autonomous Region, 2025MS03028; a project of the Department of Science and Technology of Inner Mongolia Autonomous Region, 2025YFHH0158.

## Conflicts of Interest

The authors declare that co‐author Fugui Yu is affiliated with Sunit Right Banner Jufeng Zhengbei Feed Technology Co. Ltd., a commercial feed company located at the same ranch where the experiment was conducted. This company has no commercial interests in mannitol‐based feed products.

## Data Availability

The raw sequencing data generated in this study have been deposited in the BIG Sub database of the National Genomics Data Center (NGDC), China National Center for Bioinformation, under accession number PRJCA075826. Further inquiries can be directed to the corresponding author.
